# International comparison of generic competition, prices, and usage trends: South Korea and G20 countries

**DOI:** 10.3389/fpubh.2025.1559823

**Published:** 2025-05-27

**Authors:** Da Hye Lee, Jung-Ae Kim, Kyung Min Lim, Ji Won Jung, Su Min Park, Yoon-A Jang, Jong Hyuk Lee

**Affiliations:** ^1^College of Pharmacy, Chung-Ang University, Seoul, Republic of Korea; ^2^Department of Pharmaceutical Engineering, Inje University, Gimhae-si, Republic of Korea

**Keywords:** generic drug, drug price, generic competition, drug usage, pharmaceutical expenditure, health insurance, cost savings

## Abstract

**Introduction:**

While many countries have successfully promoted generic drug use, South Korea faces challenges with low utilization and high expenditure shares, despite various policy reforms. This study aimed to evaluate the effectiveness of generic drug policies in South Korea, by comparing the prices, competition, and usage trends of generic drugs between South Korea and the Group of Twenty (G20) countries.

**Methods:**

We analyzed 26 off-patent active ingredients marketed in South Korea and G20 countries from 2014 to 2023. Generic drug prices were calculated by dividing total sales by total volume in 2023, with the average price for each country subsequently determined. Generic competition was measured by the number of generic drugs available in each country. The usage trends of generic drugs were assessed using the sales and volume ratios of off-patent original drugs to generic drugs in 2023.

**Results:**

South Korea had more generic drugs for 19 of the 26 analyzed ingredients (73.1%) compared to other G20 countries. However, for recently off-patent ingredients, the number of generic drugs was smaller in South Korea. The generic prices for 18 ingredients (69.2%) were lower in South Korea than in the G20 and Advanced Eight (A8) countries. Similar to the generic competition, generics with larger market sizes had higher prices in South Korea. Conversely, the prices of recently off-patent ingredients were higher in South Korea compared to the G20 countries. For 24 ingredients (92.3%), the sales and volume ratios of generic drugs in South Korea were considerably lower compared to the G20 countries.

**Conclusion:**

This study confirms that the pharmaceutical policies and regulatory frameworks for generic drug are fragmented and inefficient in South Korea. Consequently, these fragmented and inefficient policies disrupt the virtuous cycle mechanism of generic price and usage driven by effective competition. To address these challenges and promote the use of generic drugs, the findings of this study suggest the need to develop and implement policies in South Korea that focus on optimizing the pharmaceutical expenditure structure, enhancing post-listing price management system for generic drugs, supporting the accelerated development of generic drugs, and promoting the prescription and use of generic drugs.

## 1 Introduction

Generic drugs, with their substantially lower prices compared to original drugs, provide a cost-effective alternative that enhances patient access to healthcare and reduces government healthcare expenditures (1–4). In particular, price reductions driven by price competition among generic drugs, not only reduce the financial burden on patients and national healthcare systems but also contribute to the sustainability of health insurance finances. These savings can be reallocated to enhance other healthcare services or invested in research and development (5, 6). Given the recent global aging, the lower prices of generic drugs contribute to improving patient access to treatments for chronic diseases (5, 7, 8). Accordingly, many countries have implemented policies aimed at promoting generic drug use and stimulating price competition (9–12).

Policies to promote generic drug use vary across countries, depending on the target stakeholders, including prescribers, pharmacists, and patients. Policies targeting prescribers or pharmacists typically include prescription budget caps, mandatory prescribing using international nonproprietary name (INN), and incentive programs for prescribing or dispensing lower-cost drugs. For patients, policies commonly focus on educational programs to raise awareness of generic drugs and the implementation of a differential co-payment system to encourage the use of cost-effective options (5, 13–17). The regulatory frameworks governing generic drug pricing and stimulating price competition also differ widely across countries. The initial price of a generic drug is typically determined relative to the price of the original drug, while subsequent pricing employs diverse approaches, considering factors such market share of generic drugs, the number of generic competitors available in the market, the lowest listed price, reference pricing systems, or market-driven mechanism (15, 17–23). In countries with efficient systems, generic drug use is high, while healthcare expenditures allocated to these drugs remains low, reflecting the benefits of efficient price competition and cost containment (5, 13–17, 24, 25). According to the 2021 IQVIA report, generic drug use increased significantly from 1995 to 2020 in countries such as the United States (US), the United Kingdom (UK), Canada, and Japan. However, the increase in expenditure share remained relatively modest (26).

In contrast to the global trend, South Korea has a low rate of generic drug use and a relatively high expenditure share. In particular, because of the pharmaceutical distribution system, price reductions do not necessarily lead to increased utilization. This has driven a preference for original branded drugs among physicians, resulting in lower generic drug usage compared to that observed in major developed countries (27). Furthermore, the proliferation of generic drugs and their relatively high prices following patent expiration for original drugs have raised concerns. To address these issues, South Korea has implemented frequent policy reforms, including measures to prevent the excessive approval of generic drugs. However, few studies have evaluated the effectiveness of these policies in promoting generic drug use and stimulating price competition. Research on the optimal number and pricing of generic drugs, considering the size and structure of South Korea's pharmaceutical industry, is lacking.

This study aimed to identify the distinctive trends in South Korea's generic drug market by comparing pricing, competition, and usage trends of generic drugs with those in major international markets including the Group of Twenty (G20) and Advanced Eight (A8) countries (Japan, the US, France, Germany, Italy, Switzerland, the UK, and Canada). Additionally, we evaluated the effectiveness of policies regulating and promoting the generic drug markets in South Korea, providing insights for optimizing its future development.

## 2 Method

### 2.1 Data collection

We selected 26 off-patent active ingredients based on their patent expiration dates or market sizes to evaluate the impact of market entry timing and market size on the number of generic drugs, pricing, and usage trends. Of these, 19 active ingredients were identified based on the expiration of their substance patents between 2014 and 2023 and the availability of at least five generic drugs marketed in South Korea as of December 2023. The remaining seven active ingredients were selected based on market size, ranking among the top 50 by average sales from 2018 to 2023 in South Korea and had sales records in all G20 countries. This subsample of 26 active ingredients was specifically chosen considering that market size and patent expiration dates are recognized as key factors influencing generic drug competition, pricing, and usage trends (28–30).

For ingredients with multiple formulations and dosages, the product with the highest number of identical dosages listed within the same formulation group was selected as the representative product for analysis. Since our research did not consider product quality, which is established through bioequivalence tests, we selected representative products and compared the prices of original products, branded generics and unbranded generics. Further, the analysis excluded formulations for which price calculation was challenging, such as combination drugs or injections, non-reimbursable drugs, over-the-counter drugs, new drugs developed in South Korea, or original drugs that were not a single product.

IQVIA MIDAS sales and volume data from 2018 to 2023 was used to collect data on patent expiration dates, the number of generics marketed, annual sales and volumes, and pricing.

Among the G20 countries, Japan, the US, France, Germany, Italy, Switzerland, the UK, and Canada, which are designated as reference countries in South Korea, were categorized as A8 countries for additional analysis.

### 2.2 Data analysis

Generic competition was measured by the number of generic drugs available in each country, calculated based on the representative formulation and dosage.

For price analysis, the price per product was calculated by dividing the total sales by the total volume (number of tablets, capsules, injections, and ampoules) for 2023. The average price per ingredient in each country was then calculated for comparative analysis between South Korea and the G20 or A8 countries. Statistical differences in generic drug pricing between South Korea and the G20 or A8 countries were evaluated using a *t*-test. All statistical tests were two-sided with a significance level of 0.05.

The proportions of off-patent original and generic drugs in each country were analyzed to assess generic drug usage trends. These proportions were calculated using total sales and volume data for 2023, supporting a comparative assessment of the relative utilization of original and generic drugs across countries.

## 3 Result

Among the 26 ingredients selected for this study, 19 ingredients (73.1%) were marketed in ≥15 G20 countries. In the A8 countries, 12 ingredients (46.2%) were available in all member countries, whereas only four (15.4%) were marketed in fewer than five countries. Ingredients with larger market sizes tended to be associated with a greater number of countries where generic drugs were marketed. Atorvastatin (original brand: Lipitor Tablet 10 mg) had the most generic drugs available in South Korea. It was marketed in 19 G20 countries and all A8 countries.

In South Korea, the first approval dates of off-patent original drugs for the selected ingredients ranged widely from the early 2000s to 2014. The selected 26 ingredients, classified by Anatomical Therapeutic Chemical (ATC) codes, encompassed a broad spectrum of therapeutic categories (Table 1).

**Table 1:** Ingredients included in the analysis.

**No**.	**Ingredient name**	**Original drug**				**Market size (million USD)** ^*^			**Number of countries**		**ATC code**
		**Brand name**	**Patent expiry year**	**Approval date**	**First reimbursement date**	**Total**	**Original drug**	**Generic drugs**	**G20**	**A8**	
1	Atorvastatin^a^	Lipitor Tab. 10 mg	2008	2004-11-25	2023-01-01	1,203.8	412.6	791.3	19	8	C
2	Clopidogrel^a^	Plavix Tab. 75 mg	2007	2007-01-22	2007-03-01	1,106.6	391.5	715.1	19	8	B
3	Rosuvastatin^b^	Crestor Tab. 10 mg	2014	2002-01-15	2004-07-01	614.8	193.9	420.9	18	8	C
4	Amlodipine^a^	Norvasc Tab. 5 mg	2010	2008-12-04	2022-12-01	525.7	234.3	291.3	19	8	C
5	Tenofovir disoproxil^b^	Viread Tab. 300 mg	2018	2010-06-23	2013-11-01	390.0	347.0	43.0	18	8	J
6	Entecavir^b^	Baraclude Tab. 0.5 mg	2015	2006-05-24	2007-01-01	359.2	284.9	74.3	16	8	J
7	Donepezil^a^	Aricept Tab. 5 mg	2008	2000-08-04	2020-06-01	336.3	141.6	194.7	19	8	N
8	Celecoxib^b^	Celebrex Cap. 200 mg	2015	2006-09-11	2022-11-01	283.3	140.8	142.5	18	8	M
9	Fluconazole^a^	Diflucan Cap. 50 mg	2004	2005-07-18	2005-11-01	246.5	7.9	238.6	17	8	J
10	Montelukast^a^	Singulair Tab. 10 mg	2011	2000-11-29	2022-04-01	226.8	77.2	149.6	19	8	R
11	Dapagliflozin^b^	Forxiga Tab. 10 mg	2023	2013-11-26	2014-09-01	173.2	166.3	6.8	8	1	A
12	Apixaban^b^	Eliquis Tab. 2.5 mg	2019	2011-11-30	2013-01-01	116.4	111.5	5.0	10	2	B
13	Rivaroxaban^b^	Xarelto Tab. 15 mg	2021	2012-02-29	2013-01-01	110.7	103.6	7.1	9	0	B
14	Gefitinib^b^	Iressa Tab. 250 mg	2016	2003-06-14	2004-03-01	106.4	94.1	12.2	16	7	L
15	Pemetrexed^b^	Alimta Inj. 500 mg	2015	2005-11-30	2023-06-01	103.8	89.0	14.8	18	8	L
16	Solifenacin^b^	Vesicare Tab. 5 mg	2017	2007-03-30	2007-12-01	76.2	42.6	33.5	18	8	G
17	Metformin^a^	Glucophage Tab. 500 mg	2002	2004-03-03	2009-07-01	70.3	11.0	50.0	18	7	A
18	Oseltamivir^b^	Tamiflu Cap. 75 mg	2017	2000-06-15	2000-10-01	66.8	34.5	32.3	18	6	J
19	Bortezomib^b^	Velcade Inj. 3.5 mg	2015	2006-03-31	2016-01-01	59.0	45.7	13.4	18	7	L
20	Aripiprazole^b^	Abilify Tab. 10 mg	2014	2002-08-01	2004-02-01	58.2	45.6	12.5	17	6	N
21	Rasagiline^b^	Azilect Tab. 1 mg	2020	2013-09-13	2014-07-01	54.8	47.3	7.5	15	7	N
22	Duloxetine^b^	Cymbalta Cap. 30 mg	2014	2007-07-30	2009-04-01	47.4	26.6	20.8	17	7	N
23	Raloxifene^b^	Evista Tab. 60 mg	2015	2001-07-18	2020-08-01	39.6	27.1	12.6	14	7	G
24	Vildagliptin^b^	Galvus Tab. 50 mg	2022	2007-12-28	2009-02-01	34.5	29.8	4.6	14	5	A
25	Ticagrelor^b^	Brilinta Tab. 90 mg	2021	2011-07-22	2013-03-01	26.9	26.8	0.1	7	1	B
26	Etoricoxib^b^	Arcoxia Tab. 30 mg	2021	2014-12-23	2024-01-01	11.7	11.2	0.5	10	5	M

The results were presented according to the market size.

Tab, tablet; Cap, capsule; Inj., injection; ATC, anatomical therapeutic chemical by World Health Organization (WHO); A, alimentary tract and metabolism; B, blood and blood forming organs; C, cardiovascular system; D, dermatologicals; G, genito urinary system and sex hormones; J, antiinfectives for systemic use; L, antineoplastic and immunomodulating agents; M, musculo-skeletal system; N, nervous system; R, respiratory system; G20, group of twenty countries; A8, advanced eight countries; USD, the United State dollars.

^a^Ingredients selected based on the market size.

^b^Ingredients selected based on the patent expiry date.

^*^Market size: average sales value from 2018 to 2023 in South Korea (Data source: IQVIA MIDAS); Count as individual items, even if they have different packaging units.

Information on original drug (i.e., brand name, patient expiry year, approval date, and first reimbursement date), market size, and number of generic drugs based on South Korea.

### 3.1 International comparison of generic competition

Among the 26 ingredients, 19 ingredients (73.1%) had a higher number of generic drugs available in South Korea than in the G20 and A8 countries. South Korea has more generic drugs marketed for active ingredients with larger market sizes than G20 and A8 countries. Conversely, the number of generic drugs for seven ingredients (26.9%) with a smaller market size, including raloxifene, aripiprazole, and gefitinib, was lower in South Korea.

**Figure 1:**
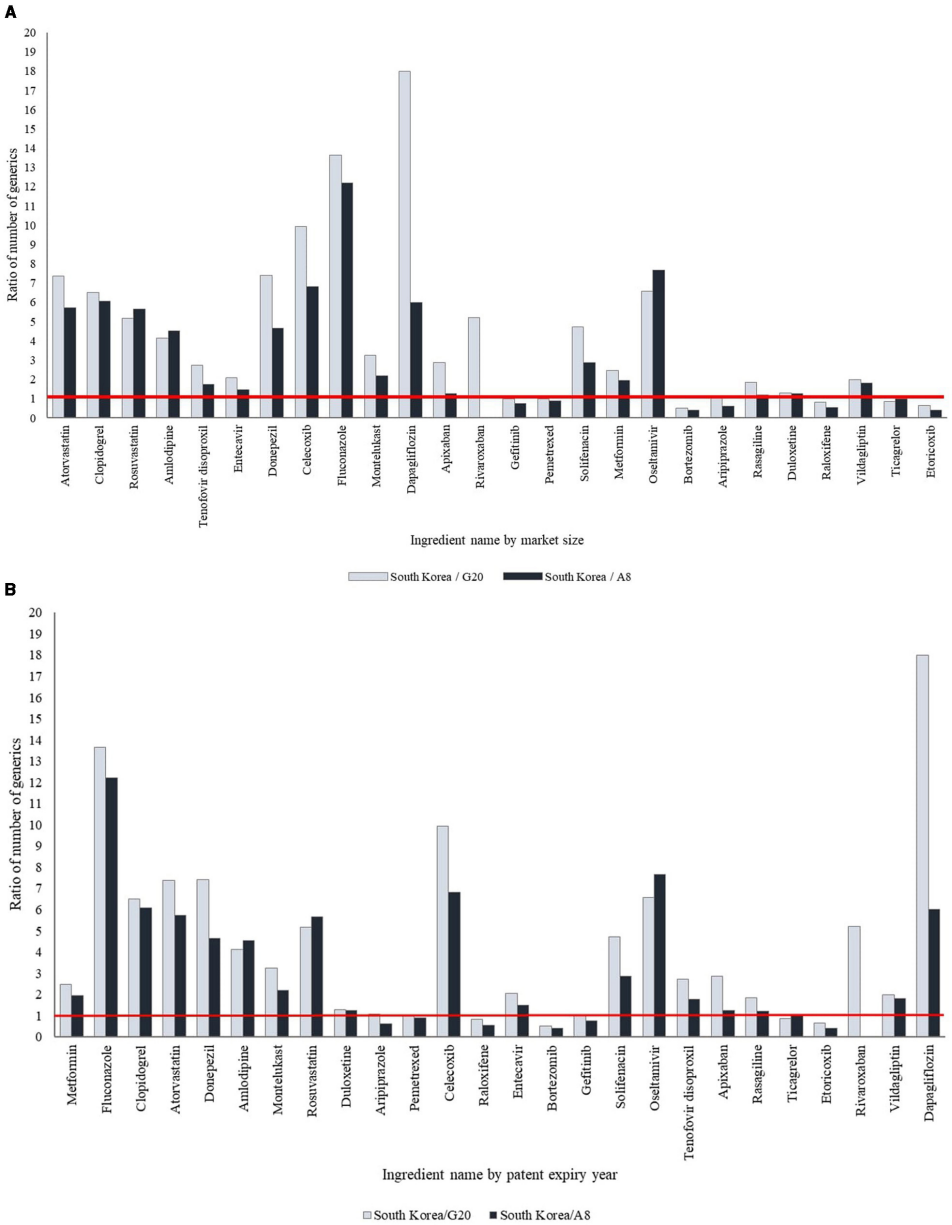
International comparison of the ratio of number of generic drugs. **(A)** By market size. The products have been arranged in order of decreasing market size. **(B)** By patent expiry year. The products have been arranged according to the year of patent expiry.

Of the 19 ingredients selected based on patent expiration dates between 2014 and 2023, 12 ingredients (63.2%) had more generics available in South Korea compared to the G20 and A8 countries, while seven (36.8%) had a comparable or lower number of generics. Recently off-patent ingredients tended to have fewer generic drugs available in South Korea than in the G20 and A8 countries, whereas ingredients with earlier patent expirations had more generics available in South Korea (Table 2, Figure 1, Supplementary Figure 1, Supplementary Table 1).

**Table 2:** International comparison of the number and prices of generic drugs.

**No**.	**Ingredient name**	**Number of generic drugs**			**Ratio of the number of generic drugs**		**Price of generic drugs**			**Ratio of the price of generic drugs**	
		**South Korea**	**G20 (average)**	**A8 (average)**	**South Korea/G20**	**South Korea/A8**	**South Korea**	**G20 (average)**	**A8 (average)**	**South Korea/G20**	**South Korea/A8**
1	Atorvastatin^a^	258	35	45	7.37	5.73	0.42	0.16^*^	0.14^*^	2.63	3.00
2	Clopidogrel^a^	267	41	44	6.51	6.07	0.72	0.35^*^	0.29^*^	2.06	2.48
3	Rosuvastatin^b^	181	35	32	5.17	5.66	0.39	0.2^*^	0.16	1.95	2.44
4	Amlodipine^a^	231	56	51	4.13	4.53	0.21	0.09^*^	0.09^*^	2.33	2.33
5	Tenofovir disoproxil^b^	30	11	17	2.73	1.76	1.56	2.65^*^	3.64^*^	0.59	0.43
6	Entecavir^b^	31	15	21	2.07	1.48	1.88	4.52^*^	6.1^*^	0.42	0.31
7	Donepezil^a^	163	22	35	7.41	4.66	1.04	0.59	0.47^*^	1.76	2.21
8	Celecoxib^b^	238	24	35	9.92	6.80	0.33	0.31^*^	0.25	1.06	1.32
9	Fluconazole^a^	232	17	19	13.65	12.21	1.11	0.8^*^	1.11	1.39	1.00
10	Montelukast^a^	94	29	43	3.24	2.19	0.49	0.34	0.33^*^	1.44	1.48
11	Dapagliflozin^b^	72	4	12	18.00	6.00	0.25	0.77^*^	0.53	0.32	0.47
12	Apixaban^b^	20	7	16	2.86	1.25	0.41	0.67^*^	0.66^*^	0.61	0.62
13	Rivaroxaban^b^	52	10	N/A	5.20	N/A	0.75	1.09^*^	N/A	0.69	N/A
14	Gefitinib^b^	6	6	8	1.00	0.75	17	37^*^	48^*^	0.46	0.35
15	Pemetrexed^b^	9	9	10	1.00	0.90	313	525	462	0.60	0.68
16	Solifenacin^b^	80	17	28	4.71	2.86	0.34	0.39^*^	0.26^*^	0.87	1.31
17	Metformin^a^	84	34	43	2.47	1.95	0.04	0.05	0.05	0.80	0.80
18	Oseltamivir^b^	46	7	6	6.57	7.67	1.1	1.52^*^	1.29	0.72	0.85
19	Bortezomib^b^	4	8	10	0.50	0.40	334	413	373	0.81	0.90
20	Aripiprazole^b^	18	17	30	1.06	0.60	0.53	0.92^*^	0.95	0.58	0.56
21	Rasagiline^b^	24	13	20	1.85	1.20	1.21	1.55^*^	1.65^*^	0.78	0.73
22	Duloxetine^b^	30	23	24	1.30	1.25	0.23	0.35	0.27^*^	0.66	0.85
23	Raloxifene^b^	9	11	17	0.82	0.53	0.46	0.57	0.53	0.81	0.87
24	Vildagliptin^b^	18	9	10	2.00	1.80	0.15	0.36^*^	0.34^*^	0.42	0.44
25	Ticagrelor^b^	6	7	6	0.86	1.00	0.36	0.53^*^	0.38	0.68	0.95
26	Etoricoxib^b^	7	11	17	0.64	0.41	0.18	0.27^*^	0.32^*^	0.67	0.56

The results were presented according to the market size.

G20, group of twenty countries; A8, advanced eight countries; N/A, not available.

^a^Ingredients selected based on the market size.

^b^Ingredients selected based on the patent expiry date.

p-value was derived from t-test.

^*^p-value < 0.05.

### 3.2 International comparison of generic price

**Figure 2:**
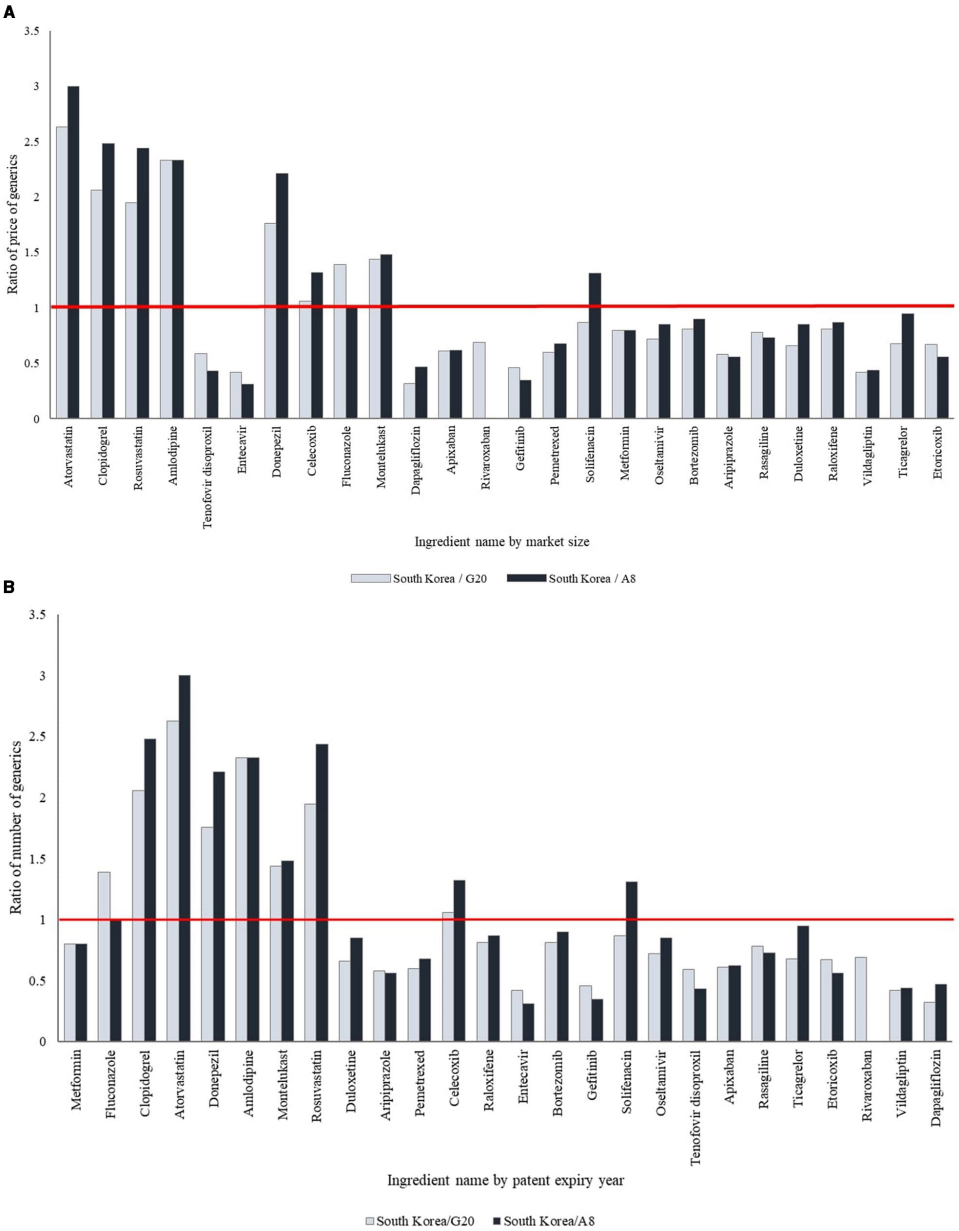
International comparison of the ratio of price of generic drugs. **(A)** By market size. The products have been arranged in order of decreasing market size. **(B)** By patent expiry year. The products have been arranged according to the year of patent expiry.

Of the 26 selected ingredients, eight ingredients (30.8%), including donepezil, montelukast, and rosuvastatin, had significantly higher generic prices in South Korea compared to the G20 and A8 countries, whereas 14 (53.8%) showed significantly lower prices (*p* < 0.05). For the remaining four ingredients (5.4%), the generic prices were lower in South Korea, though the differences were not statistically significant. The prices of generic drugs in South Korea showed variation based on market size, with ingredients having larger market sizes generally presenting higher prices than those with smaller market sizes. Among the 19 ingredients with patents expiring between 2014 and 2023, two ingredients (10.5%) had higher generic prices in South Korea compared to the G20 and A8 countries, whereas 17 (89.5%) had lower generic prices. Moreover, recently off-patent ingredients had lower generic prices in South Korea compared to the G20 and A8 countries, whereas those with earlier patent expirations had higher prices (Table 2, Figure 2, Supplementary Table 1).

### 3.3 International comparison of generic usage trends

Across the 26 ingredients analyzed in this study, 24 ingredients (92.3%) showed a lower proportion of generic drug sales and usage in South Korea compared to those observed in the G20 and A8 countries. Ingredients with larger market sizes or earlier patient expiration dates showed higher sales and usage ratios for generic drugs in South Korea (Table 3).

**Table 3:** International comparison of the ratio of sales and volume of original drugs and generic drugs.

**No**.	**Ingredient name**	**South Korea**		**G20 (average)**		**A8 (average)**	
		**Sales (O:G)**	**Volume (O:G)**	**Sales (O:G)**	**Volume (O:G)**	**Sales (O:G)**	**Volume (O:G)**
1	Atorvastatin^a^	34%:66%	34%:66%	27%:73%	14%:86%	30%:70%	9%:91%
2	Clopidogrel^a^	33%:67%	35%:65%	25%:75%	18%:82%	30%:70%	9%:91%
3	Rosuvastatin^b^	30%:70%	30%:70%	34%:66%	16%:84%	29%:71%	4%:96%
4	Amlodipine^a^	43%:57%	41%:59%	24%:76%	11%:89%	19%:81%	8%:92%
5	Tenofovir disoproxil^b^	86%:14%	86%:14%	30%:70%	18%:82%	23%:77%	11%:89%
6	Entecavir^b^	76%:24%	75%:25%	30%:70%	16%:84%	32%:68%	20%:80%
7	Donepezil^a^	36%:64%	31%:69%	39%:61%	10%:90%	43%:57%	9%:91%
8	Celecoxib^b^	46%:54%	46%:54%	41%:59%	30%:70%	31%:69%	18%:82%
9	Fluconazole^a^	3%:97%	4%:96%	28%:72%	21%:79%	27%:73%	23%:77%
10	Montelukast^a^	28%:72%	28%:72%	19%:81%	13%:87%	16%:84%	10%:90%
11	Dapagliflozin^b^	84%:16%	75%:25%	87%:13%	85%:15%	99%:1%	97%:3%
12	Apixaban^b^	100%:0%	100%:0%	64%:36%	58%:42%	37%:63%	29%:71%
13	Rivaroxaban^b^	72%:28%	70%:30%	67%:38%	57%:43%	N/A	N/A
14	Gefitinib^b^	81%:19%	79%:21%	52%:48%	38%:62%	42%:58%	29%:71%
15	Pemetrexed^b^	85%:15%	84%:16%	23%:77%	17%:83%	24%:76%	15%:85%
16	Solifenacin^b^	46%:54%	46%:54%	37%:63%	26%:74%	36%:64%	20%:80%
17	Metformin^a^	21%:79%	16%:84%	36%:64%	22%:78%	9%:91%	6%:94%
18	Oseltamivir^b^	70%:30%	69%:31%	49%:51%	41%:59%	49%:51%	43%:57%
19	Bortezomib^b^	88%:12%	88%:12%	15%:85%	8%:92%	18%:82%	8%:92%
20	Aripiprazole^b^	78%:22%	52%:48%	22%:78%	12%:88%	13%:87%	8%:92%
21	Rasagiline^b^	63%:37%	63%:37%	44%:56%	38%:62%	37%:63%	27%:73%
22	Duloxetine^b^	55%:45%	48%:52%	22%:78%	13%:87%	26%:74%	16%:84%
23	Raloxifene^b^	58%:42%	59%:41%	38%:62%	33%:67%	21%:79%	16%:84%
24	Vildagliptin^b^	50%:50%	52%:48%	64%:36%	58%:42%	59%:41%	50%:50%
25	Ticagrelor^b^	97%:3%	97%:3%	69%:31%	57%:43%	38%:62%	17%:83%
26	Etoricoxib^b^	73%:27%	72%:28%	50%:50%	46%:54%	36%:64%	30%:70%

The results were presented according to the market size.

G20, group of twenty countries; A8, advanced eight countries; N/A, not applicable; O, original drugs; G, generic drugs.

^a^Ingredients selected based on the market size.

^b^Ingredients selected based on the patent expiry date.

## 4 Discussion

This study found that both the price and number of generic drugs in South Korea varied according to market size and the timing of patent expiration. Among the 26 ingredients analyzed, 19 had a higher number of generic drugs in South Korea compared to other G20 countries, while recently off-patent ingredients showed fewer generics. In terms of pricing, generic drugs for 18 out of the 26 ingredients were priced lower in South Korea; however, for the recently off-patent ingredients, generic prices in South Korea were higher than those in the G20 countries. Additionally, the sales and volume ratios of generic drugs in South Korea were considerably lower compared to those in the G20 countries.

Regulatory changes, including the introduction of “1+3 Bioequivalence Policy” in 2020 and the “Differential Generic Pricing System” in 2021, have likely curbed the number of generics launched in recent years. The “1+3 Bioequivalence Policy” restricts market approvals to a maximum of three generic products that may be approved by using previously submitted bioequivalence (BE) test or clinical trial data. The “Differential Generic Pricing System” differentiates generic prices based on whether bioequivalence or clinical studies were conducted by a generic manufacturer and whether an active pharmaceutical ingredient (API) used is registered in the Drug Master File (DMF) of the Ministry of Food and Drug Safety (MFDS). According to this system, the generic price is set at 53.55% of the original price when both criteria are met; if only one criterion is met, the price is set at 45.52% of the original price; and when neither criterion is met, the price is set at 38.69% of the original price. These measures were implemented to address the issue of an excessive number of identical generic drugs entering the market. As a result, these policies are presumed to have reduced the number of generics launched in recent years compared to earlier periods.

An excessive number of generic drugs for a single ingredient may lead to challenges associated with market overcrowding, while a limited number of generics could result in drug supply shortages, posing another societal issue. Therefore, it is important to maintain an optimal balance in the number of generic drugs. Since the implementation of the “1+3 Bioequivalence System” and the “Differential Generic Pricing System,” the cases where more than 100 generic drugs are marketed has significantly decreased, demonstrating the effectiveness of these policies. However, certain ingredients still show an excessive number of marketed generics. This highlights the need for regulatory measures to manage the optimal number of generic drugs. Additionally, government support is crucial for the development of generic drugs, particularly for products that encounter substantial formulation or manufacturing challenges.

In the early stages following patent expiration, generic drug prices in South Korea are set at considerably lower levels compared to those in major foreign countries. Generic drug prices in these foreign markets, however, continue to decline progressively over time, eventually resulting in a reversal of the price trend (5). Given this trend, which reflects the fundamental characteristics of South Korea's drug pricing system, a comparison of generic drug prices at specific time points indicates that prices in South Korea tend to be higher than those in other major countries.

Therefore, it may not be appropriate to reduce prices solely based on higher observed levels compared to other countries. Alternatively, it is crucial to consider the substantial financial savings achieved during the initial generic listing when referencing foreign prices to reevaluate and adjust drug prices. This suggests the need to implement mechanisms that incorporate these initial savings into the post-listing price management system to improve policy effectiveness and ensure sustainability.

The study also highlighted lower proportions of generic drug sales and usage in South Korea compared to other major markets. Over many years, South Korea has implemented policies to foster a generic-focused pharmaceutical industry. As a result, a pharmaceutical expenditure spending structure has been established, ensuring that the proportion of spending on generic drugs remains above a certain level, in alignment with this industrial strategy. To modernize the pharmaceutical expenditure structure, it is imperative to consider not only the trends in pharmaceutical spending observed in advanced global markets, but also the structure of South Korea's pharmaceutical industry. South Korea pharmaceutical distribution system does not inherently support an increased use driven by market dynamics following price reductions, unlike advanced markets. This suggests that the issue may lie in how price reductions are implemented with the market. Specially, it may mean that the entire burden of price reductions translates into financial losses for pharmaceutical companies rather than leading to increased market uptake. Therefore, it is important to reform the expenditure structure by considering the financial contributions of generic drugs and the unique dynamics of South Korea's pharmaceutical industry, rather than reducing the prices of generic drugs due to their high proportion of pharmaceutical expenditures. Implementing price reductions without policies to promote the utilization of generic drugs is misaligned with the objectives of improving the expenditure structure. Additionally, physician preferences for original branded drugs remain stronger in South Korea compared to major developed countries, further hindering the utilization of generis. In light of this, policies that promote the use of generic drugs should be prioritized to enhance the expenditure structure and ensure that price reductions translate into increased utilization.

The “Low-Cost Purchasing Initiatives” system and the “Low-Cost Drug Substitution Incentive Program” were introduced to encourage the prescription of lower-cost drugs, including generic drugs. According to the Ministry of Health and Welfare, the Low-Cost Purchase Initiatives system has resulted in fiscal savings of 100–200 billion KRW annually from 2015 to 2022 (31). However, while the policy has demonstrated fiscal savings, its impact on the use of generic drugs has not been adequately evaluated. As such, although these polices have contributed to fiscal savings, their effectiveness in encouraging the use of generic drugs remains uncertain. This highlights the need for further reforms that not only support price reductions but also incentivize the broader use of generics. This would contribute to the long-term sustainability of South Korea's pharmaceutical expenditure structure.

In South Korea, drug prices are continuously reduced through various post-listing price management systems after reimbursement listing (32–34). This approach provides the benefit of achieving prompt cost savings for the health insurance system, however, it primarily targets generic drugs. Therefore, policy decisions should consider the potential impact on patient access to treatment and the pharmaceutical industry. In particular, the continuous reduction in the prices of generic drugs may lead to shortages of essential medicines, potentially impeding patient access to treatment. Additionally, South Korea's post-listing price management system has been criticized for being inefficient due to the complex interaction of various policies. The fragmentation of these systems and the frequent, unpredictable reduction of drug prices are primary factors underlying low predictability, leading to issues such as confusion among healthcare institutions due to price discrepancies. Moreover, unpredictable price reductions through post-listing management systems focused solely on cost-saving, irrespective of drug listing principles, may conflict with the pharmaceutical industry's advancement. Even for generic drugs that substantially contribute to cost savings, prices are reduced under the volume-based pricing system solely due to increased usage. This highlights the need to reconsider whether such measures align with the policy's objectives.

Considering global trends in pharmaceutical expenditure structures, promoting the use of generic drugs is essential not only for achieving cost savings but also as a fundamental step toward reforming pharmaceutical expenditure frameworks. However, South Korea lacks effective systems to encourage generic drug utilization, and the current policies are ineffective.

In South Korea, reimbursement prices for generic drugs are determined by a pricing formula, which lists up to 20 generics meeting specific criteria at the same price as both the original drug and other generics (35, 36). Under this system, companies are discouraged from lowering prices, as price reductions do not result in increased usage. Moreover, lower-priced transactions further drive down prices, causing financial losses for pharmaceutical companies. Therefore, it suggests that the system should be reformed to ensure pharmaceutical companies do not incur financial losses during low-priced transactions and to promote the use of generic drugs through market-driven competition. Additionally, the lack of price competition in the distribution system drives a marked preference among physicians for originator drugs, leading to substantially lower utilization of generics compared to other major developed countries (27). It is imperative to establish policy mechanisms that promote competitive dynamics among generic drugs, ultimately driving their increased utilization. These measures should be structured to ensure tangible advantages for physicians, pharmacists, and patients using generics, thereby facilitating their broader adoption.

Despite the comparative insights into South Korea's policy effectiveness and its future development, several limitations should be taken into consideration when interpreting the results. First, this study conducted a descriptive analysis to compare the usage trend of generic drugs across countries without accounting for factors that may influence these trends, such as the timing of the first generic listing, demand for alternative drugs, and price advantages. In particular, the analysis was limited to the recent 5 years due to constraints in the available sales data. Also, no attempt was made to match the product sample in terms of the quality of generic products, which is established through bioequivalence tests. Therefore, it is possible that some of the variation in prices and, hence, price ratios may reflect differences in product quality. Further studies could be considered to explore the impact of these factors. Second, due to the inherent limitations of IQVIA MIDAS database, ingredients with different packaging unit were considered as individual generic drug. However, it was confirmed that the type and numbers of packaging units were consistent across the selected countries, suggesting that the impact of packaging unit variations was unlikely to significantly affect the results of this study.

## 5 Conclusion

Promoting the utilization of generic drugs not only helps address supply shortages by ensuring a stable supply of medicines but also contributes to financial sustainability through health cost savings, improving patient access to medications, and advancing the pharmaceutical industry.

However, South Korea's policies and regulatory frameworks governing generic drug approval, pricing, post-listing price management, and utilization are fragmented and inefficient, which consequently leads to the disruption of the mechanism that facilitates price reductions and increased utilization through effective competition. Generic drug prices in South Korea are initially set lower than in other countries, but their subsequent price reductions over time are not as substantial as those observed in other countries. Moreover, it has been observed that the usage rates of off-patent original drugs in South Korea remain significantly higher than in other countries, even after patent expiration. To address these challenges and promote the use of generic drugs, the findings of this study suggest the need for the development and implementation of comprehensive policies in South Korea. These policies should aim to optimize pharmaceutical expenditure structure, reform the post-listing price management system for generic drugs, accelerate the development and market entry of generic drugs, and promote their prescription and use.

## Data Availability

The raw data supporting the conclusions of this article will be made available by the authors, without undue reservation.
