# Supplementary material for: International comparison of generic competition, prices, and usage trends: South Korea and G20 countries
Source: Front Public Health. 2025 May 27;13:1559823. doi: 10.3389/fpubh.2025.1559823 (PMC12150593; doi:10.3389/fpubh.2025.1559823)
Supplement: Supplementary file 1 [file Table_1.docx]

**Supplementary Table 1. International comparison of the number and prices of generic drugs**

| **No.** | **Ingredient name** | **Number of generic drugs** | | |  | **Ratio of the number of generic drugs** | |  | **Price of generic drugs** | | |  | **Ratio of the price of**  **generic drugs** | |
| --- | --- | --- | --- | --- | --- | --- | --- | --- | --- | --- | --- | --- | --- | --- |
|  |  | **South Korea** | **G20 (Average)** | **A8 (Average)** |  | **South Korea**  **/G20** | **South Korea**  **/A8** |  | **South Korea** | **G20 (Average)** | **A8 (Average)** |  | **South Korea**  **/G20** | **South Korea**  **/A8** |
| 1 | Metformin^2^ | 84 | 34 | 43 |  | 2.47 | 1.95 |  | 0.04 | 0.05 | 0.05 |  | 0.8 | 0.8 |
| 2 | Fluconazole^2^ | 232 | 17 | 19 |  | 13.65 | 12.21 |  | 1.11 | 0.8^*^ | 1.11 |  | 1.39 | 1 |
| 3 | Clopidogrel^2^ | 267 | 41 | 44 |  | 6.51 | 6.07 |  | 0.72 | 0.35^*^ | 0.29^*^ |  | 2.06 | 2.48 |
| 4 | Atorvastatin^2^ | 258 | 35 | 45 |  | 7.37 | 5.73 |  | 0.42 | 0.16^*^ | 0.14^*^ |  | 2.63 | 3 |
| 5 | Donepezil^2^ | 163 | 22 | 35 |  | 7.41 | 4.66 |  | 1.04 | 0.59 | 0.47^*^ |  | 1.76 | 2.21 |
| 6 | Amlodipine^2^ | 231 | 56 | 51 |  | 4.13 | 4.53 |  | 0.21 | 0.09^*^ | 0.09^*^ |  | 2.33 | 2.33 |
| 7 | Montelukast^2^ | 94 | 29 | 43 |  | 3.24 | 2.19 |  | 0.49 | 0.34 | 0.33^*^ |  | 1.44 | 1.48 |
| 8 | Rosuvastatin^1^ | 181 | 35 | 32 |  | 5.17 | 5.66 |  | 0.39 | 0.2^*^ | 0.16 |  | 1.95 | 2.44 |
| 9 | Duloxetine^1^ | 30 | 23 | 24 |  | 1.3 | 1.25 |  | 0.23 | 0.35 | 0.27^*^ |  | 0.66 | 0.85 |
| 10 | Aripiprazole^1^ | 18 | 17 | 30 |  | 1.06 | 0.6 |  | 0.53 | 0.92^*^ | 0.95 |  | 0.58 | 0.56 |
| 11 | Pemetrexed^1^ | 9 | 9 | 10 |  | 1 | 0.9 |  | 313 | 525 | 462 |  | 0.6 | 0.68 |
| 12 | Celecoxib^1^ | 238 | 24 | 35 |  | 9.92 | 6.8 |  | 0.33 | 0.31^*^ | 0.25 |  | 1.06 | 1.32 |
| 13 | Raloxifene^1^ | 9 | 11 | 17 |  | 0.82 | 0.53 |  | 0.46 | 0.57 | 0.53 |  | 0.81 | 0.87 |
| 14 | Entecavir^1^ | 31 | 15 | 21 |  | 2.07 | 1.48 |  | 1.88 | 4.52^*^ | 6.1^*^ |  | 0.42 | 0.31 |
| 15 | Bortezomib^1^ | 4 | 8 | 10 |  | 0.5 | 0.4 |  | 334 | 413 | 373 |  | 0.81 | 0.9 |
| 16 | Gefitinib^1^ | 6 | 6 | 8 |  | 1 | 0.75 |  | 17 | 37^*^ | 48^*^ |  | 0.46 | 0.35 |
| 17 | Solifenacin^1^ | 80 | 17 | 28 |  | 4.71 | 2.86 |  | 0.34 | 0.39^*^ | 0.26^*^ |  | 0.87 | 1.31 |
| 18 | Oseltamivir^1^ | 46 | 7 | 6 |  | 6.57 | 7.67 |  | 1.1 | 1.52^*^ | 1.29 |  | 0.72 | 0.85 |
| 19 | Tenofovir disoproxil^1^ | 30 | 11 | 17 |  | 2.73 | 1.76 |  | 1.56 | 2.65^*^ | 3.64^*^ |  | 0.59 | 0.43 |
| 20 | Apixaban^1^ | 20 | 7 | 16 |  | 2.86 | 1.25 |  | 0.41 | 0.67^*^ | 0.66^*^ |  | 0.61 | 0.62 |
| 21 | Rasagiline^1^ | 24 | 13 | 20 |  | 1.85 | 1.2 |  | 1.21 | 1.55^*^ | 1.65^*^ |  | 0.78 | 0.73 |
| 22 | Ticagrelor^1^ | 6 | 7 | 6 |  | 0.86 | 1 |  | 0.36 | 0.53^*^ | 0.38 |  | 0.68 | 0.95 |
| 23 | Etoricoxib^1^ | 7 | 11 | 17 |  | 0.64 | 0.41 |  | 0.18 | 0.27^*^ | 0.32^*^ |  | 0.67 | 0.56 |
| 24 | Rivaroxaban^1^ | 52 | 10 | N/A |  | 5.2 | N/A |  | 0.75 | 1.09^*^ | N/A |  | 0.69 | N/A |
| 25 | Vildagliptin^1^ | 18 | 9 | 10 |  | 2 | 1.8 |  | 0.15 | 0.36^*^ | 0.34^*^ |  | 0.42 | 0.44 |
| 26 | Dapagliflozin^1^ | 72 | 4 | 12 |  | 18 | 6 |  | 0.25 | 0.77^*^ | 0.53 |  | 0.32 | 0.47 |

Note: The results were presented according to patent expiry year

Abbreviations: G20, Group of Twenty Countries; A8, Advanced Eight Countries; N/A, Not Available.

^1^ Ingredients selected based on the patent expiry date;^2^ Ingredients selected based on the market size.

p-value was derived from t-test; * p-value < 0.05
