# Supplementary material for: International comparison of generic competition, prices, and usage trends: South Korea and G20 countries
Source: Front Public Health. 2025 May 27;13:1559823. doi: 10.3389/fpubh.2025.1559823 (PMC12150593; doi:10.3389/fpubh.2025.1559823)

Supplementary Figure 1. Trends in the number of generic drugs in South Korea

(A) By market size

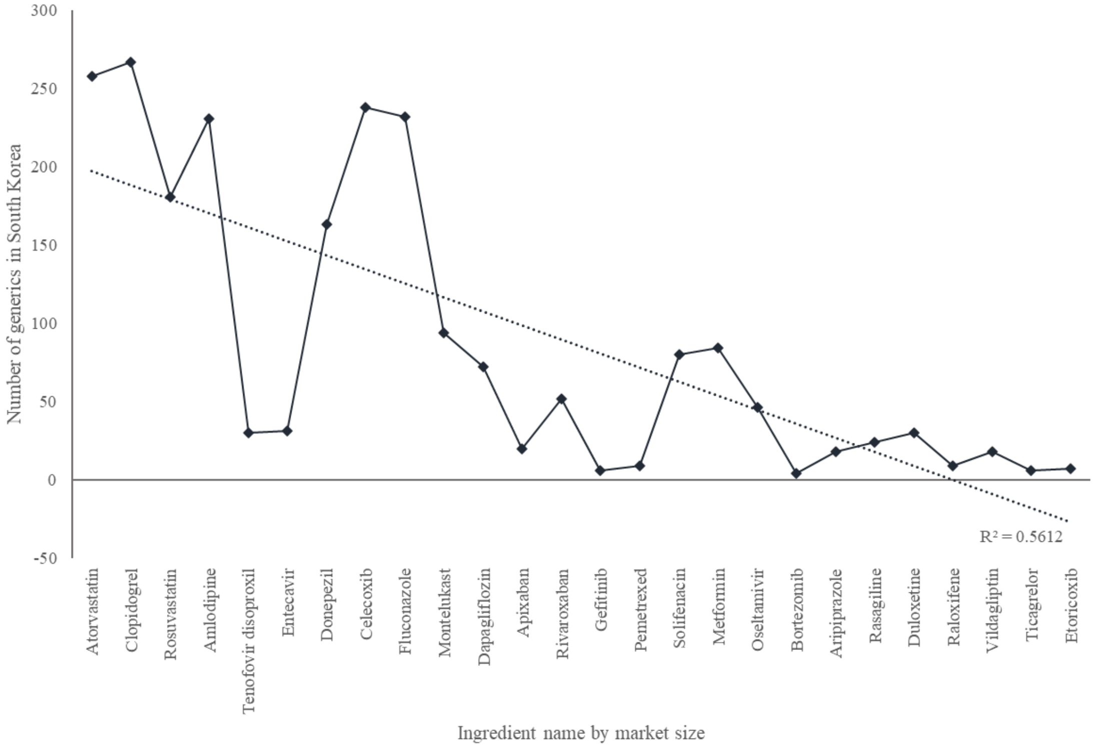

(B) By patent expiry year

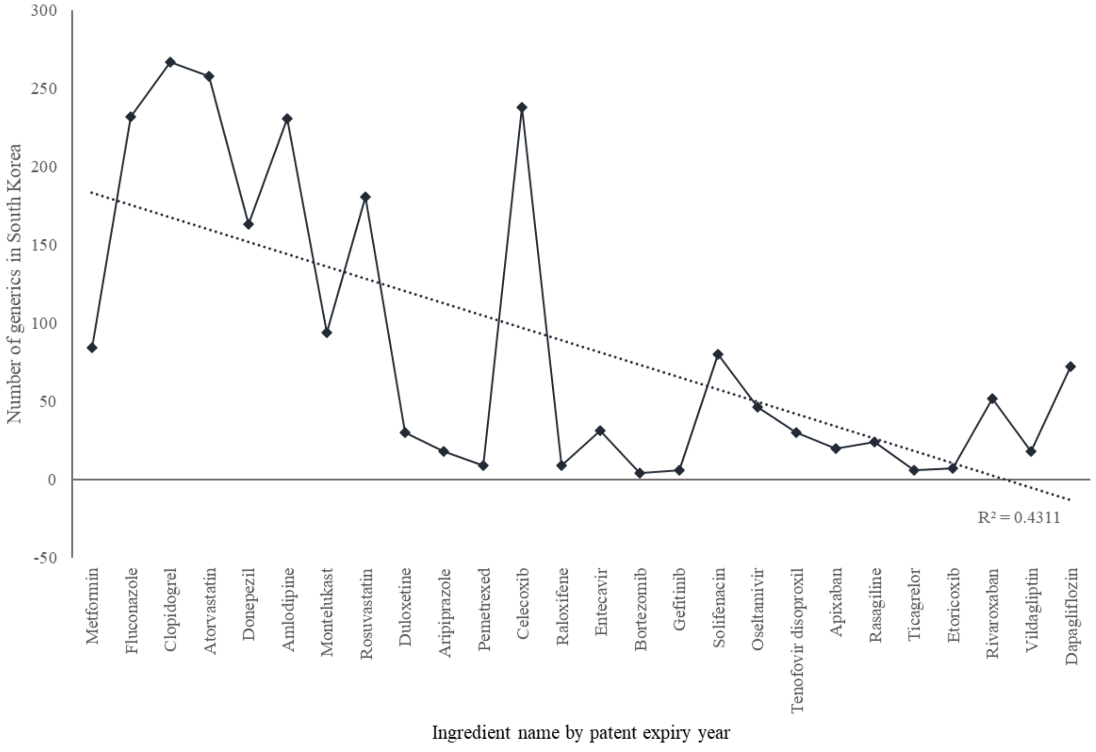

Supplement: Supplementary file 2 [file Data_Sheet_1.pdf]
